# Three-dimensional X-ray thermography using phase-contrast imaging

**DOI:** 10.1038/s41598-018-30443-4

**Published:** 2018-08-23

**Authors:** Akio Yoneyama, Akiko Iizuka, Tatsuo Fujii, Kazuyuki Hyodo, Jun Hayakawa

**Affiliations:** 1 Research and Development Group, Hitachi Ltd., 1-280 Higashi-koigakubo, Kokubunji, 185-8601 Japan; 2 Research and Development Group, Hitachi Ltd., 832-2 Horiguchi, Hitachinaka, Ibaraki, 312-0034 Japan; 3 High Energy Accelerator Research Organization, 1-1 Oho, Tsukuba, Ibaraki, 305-0801 Japan

## Abstract

Thermal management is a key technology to desterilize unused energy sources for building sustainable societies. However, conventional temperature measurement methods such as infrared thermography can detect only the surface temperature of objects because they use infrared light. We thus present a novel three-dimensional X-ray thermography using a phase-contrast X-ray imaging technique, which enables non-destructive observations of the inner thermal distribution of samples. The sensitivity of phase-contrast X-ray imaging is about 1000 times higher than that of conventional X-ray imaging. Therefore, temperature changes can be detected by using density changes caused by thermal expansion. We applied X-ray interferometric imaging (XI) that detects phase-shift by using a crystal X-ray interferometer. The highest sensitivity of XI was utilized to successfully obtain the first three-dimensional image that visualizes the thermal distribution in heated water nondestructively. Additionally, projection images visualizing the dynamic thermal flow in heated water were also obtained, and their distribution and diffusion velocity agreed well with those of the calculated images obtained by computational fluid dynamics analysis. These results show that the novel thermography enables nondestructive observations of inner temperature and thermal flow and can provide solutions for optimum thermal design of electrical devices, motors, and engines.

## Introduction

Along with length and weight, temperature is one of the basic physical values of materials, which expresses the thermal energy generated by the vibration of atoms and molecules. Therefore, thermal management is an important key technology to desterilise unused energy sources for building a sustainable society. Temperature of an object can be measured using thermistors, resistive temperature detectors, thermocouples, infrared thermography, or pyrometers. However, these devices detect the temperature directory through physical contact or using infrared and visible light from the object, and therefore, the detectable depth is limited to a few mm from its surface. So, its inner temperature distribution cannot be measured in principle. Alternatively, temperature can also be detected from lattice vibrations of a sample using X-ray diffraction indirectly, but this method is limited to crystalline materials. Thermal expansion (density change) caused by increasing temperatures could be used to detect temperature. However, the change in density is very small (<1e^−5^/K), and therefore, it cannot be detected by using conventional X-ray computed tomography (CT) because of its low sensitivity for density.

Recently, phase-contrast X-ray imaging using phase-shift information of a passing sample has been actively developed^1–7^. The sensitivity for electron density is about 1000 times higher than that of conventional X-ray CT for light elements, and enables fine and precise observations of biomedical and material samples mainly consisting of light elements such as oxygen, nitrogen, and carbon. X-ray interferometric (XI) phase-contrast imaging^1^, which detects phase-shift using a crystal X-ray interferometer, has the highest sensitivity compared with that of other phase-sensitive methods such as diffraction-enhanced imaging and the Talbot interferometric method^8,9^, because it detects the phase-shift directly from the superposition of X-rays. This high-sensitivity was utilized to clearly visualise, β-amyloid plaques in brains taken from Alzheimer disease mice models^10^, cancerous tissues in rat livers^11^, and air hydrates in old ice cores mined from Antarctica^12^ without any contrast agents or harmful X-ray doses. In addition, dynamic observation of cations and anions in the electrolytic solution of a lithium-ion battery cell was also performed successfully during its discharge and charge cycle^13^.

**Figure 1:**
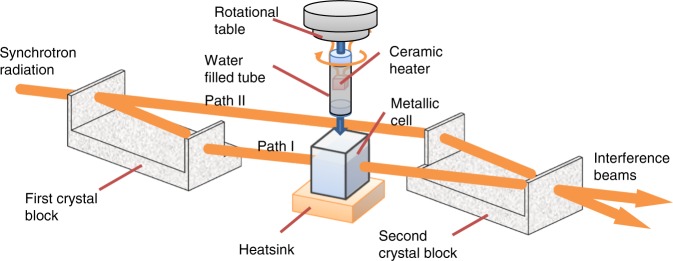
Schematic view of the XI system and demonstrative sample. The X-ray interferometer is composed of two-crystal blocks with two thin wafers acting as X-ray half mirrors, so the incident X-ray was divided, refract, and combined to generate two interference beams. Phase-shift caused by water in the tube placed in path I was detected by the intensity changes of the interference beams. The whole tube was rotated in a metallic cell filled with water cooled by a heatsink to perform computed tomography.

The coefficient of the thermal linear expansion of water and iron are 70 and 12 × 10^−6^/K, respectively, and therefore, the high sensitivity of XI enables us to observe two- and three-dimensional temperature maps (spatial distribution) of a sample by means of density changes. A density resolution of 0.5 mg/cm^3^ was attained for an XI imaging system using feasibility observations with a 1-hour measurement time^14,15^ and monochromated synchrotron radiation X-rays emitted from a vertical wiggler at the beamline BL-14C of the Photon Factory (synchrotron facility) in Japan. As shown in equation (6) in the supplemental method, the temperature changes can be obtained from the measured phase-shift directly, and the thermal detection limit (temperature resolution) is estimated as 2 and 6 degrees for water and iron, respectively, from the density resolution. A three-dimensional feasibility observation was performed using a polypropylene tube (10-mm diameter) filled with water heated by a ceramic heater attached to the upper inside of the tube. To obtain phase-contrast computed tomography data^2^, the whole tube is rotated by a motor inside of an outer metallic cell, which was filled with water cooled by a heatsink. The outer cell is placed in the path of the X-ray interferometer beam as shown in Fig. 1. The heater operates at 5.3 W during the three-dimensional measurement period (50 min), and the thermal condition of the water in the tube is kept constant.

**Figure 2:**
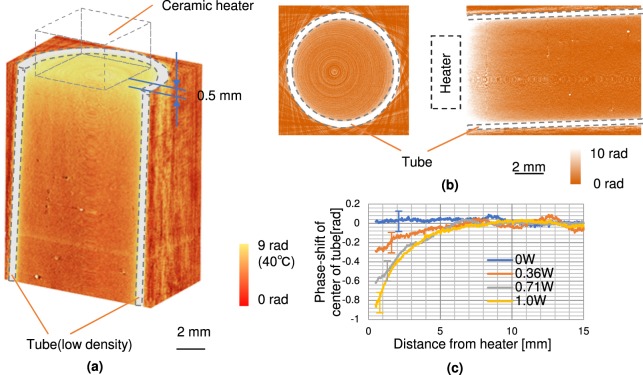
Three-dimensional thermal image of heated water in tube (**a**), expansion images in axial and sectional direction (**b**), and line profiles of phase-shift at centre of tube in sagittal direction (**c**). The temperature upper area near heater was higher than bottom area in images (**a**) and (**b**). The highest temperature was proportional to heating power, and temperature changed in an exponential manner in (**c**).

An obtained three-dimensional volume rendering image of the thermal distribution of the heated water is shown in Fig. 2(a). The heater was positioned 0.5 mm above the rendered image, as indicated by the wireframe box. The front half region of the tube was cut to show the inner temperature. The result shows that the temperature in the upper region near the heater is at its highest (white), decreasing as the distance from the heater increases (transitioning to red). Note that the temperature of the tube appears to be higher than that of the heated center area because its density is lower than that of water, as indicated by the wireframe outlines.

Figure 2(b) shows expansion images in the axial direction at 1 mm from the heater (left) and sagittal (vertical) direction from the center of the tube (right). The axial direction image shows that the temperature is almost the same value in the centre and at the edge. The phase difference between the top and bottom areas is about 9 rad (δ = 1.0 × 10^−7^) in the sagittal image, which corresponds to 40 degrees. The temperature resolution limitation calculated from the standard deviation of the phase fluctuation in a background area is about 2 degrees.

The line profiles in Fig. 2(c) shows the phase-shift at the centre of the tube in the sagittal direction obtained under different heating power levels (0, 0.36, 0.7, and 1.0 W). Each phase-shift at the farthest point from the heater is set to 0 degrees. The temperatures near the heater are increased proportionally to the heating power level, and its profile forms an exponential shape that is generally expected from the thermal diffusion equation.

**Figure 3:**
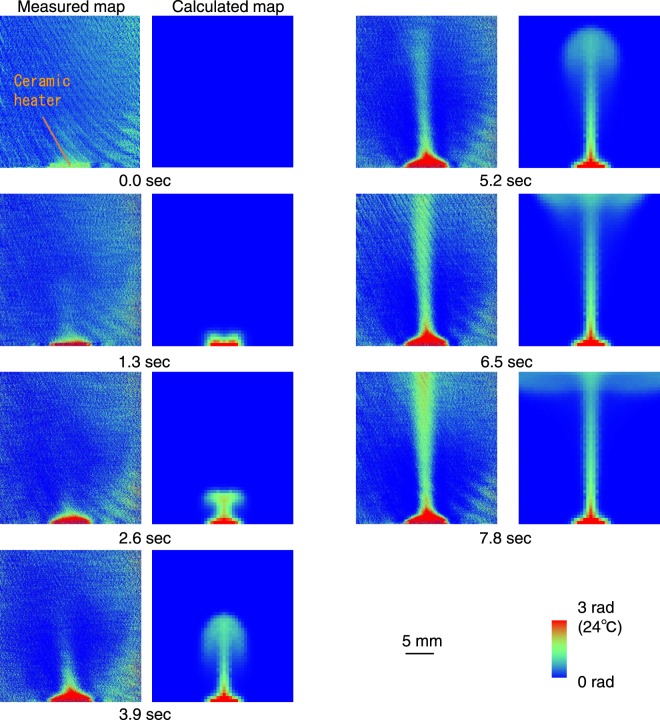
Time-resolved projection images of thermal flow in heated water filled cell ((left) measured map and (right) calculated map). High temperature region (white) expanded to upper area as columnar shape. Thermal spatial distribution and diffusion velocity of calculated map agrees well with measured map.

A time-resolved observation of heated water in an acrylic cuboid cell (10 mm thick) was also performed using the same XI imaging system as a demonstration to visualise dynamic thermal flow. Phase-contrast projection images are obtained in continuity while water is heated by the ceramic heater. Figure 3 shows a series of time-resolved projection map image pairs of thermal flow taken at 1.3-sec intervals. The left images show the measured map of thermal distribution after heating started, and the right images show the calculated maps of thermal distribution by finite element method with the same conditions (heater power, thick ness of cell and water, surrounding conditions and initial temperature. Calculation interval (temporal resolution) was 0.1 sec.). By switching on the heater, the temperature near the heater increased gradually (1.3-sec maps), and after a few seconds, a high temperature region (grey) expands to the upper area as columnar shape. The highest temperature recorded near the heater was 40 degrees, which coincides with that obtained from conventional infrared thermography. The images calculated by computational fluid dynamics analysis using STAR CCM+ show the same thermal distribution and diffusion rate, so X-ray thermography observes the thermal phenomenon correctly. Note that the ceramic heater placed at the bottom of the cell is not transparent enough for phase-shift to be detected, and therefore its density could not be detected correctly and the contrast is mixed. The distribution of the hot area in the measured maps was blurred comparing with that of calculated map, because the time-resolution of the measurement was 1.3 sec.

**Figure 4:**
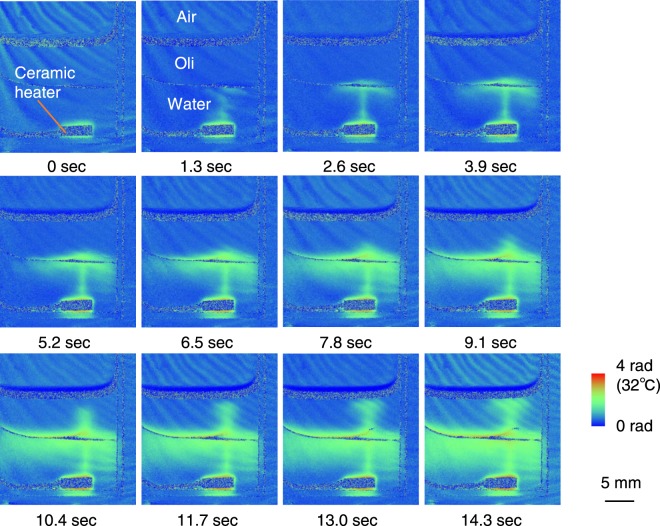
Time-resolved projection images of thermal flow in two-layered liquid (water and machine oil). After reaching the top surface of the water, the high temperature region spread not only horizontally but also vertically, and the oil was heated by the water.

Figure 4 shows a series of images of the thermal flow in a two-layer liquid (water and machine oil) taken under the same conditions as those shown in Fig. 3. Because water and oil are immiscible, no separation material between the liquids such as an acrylic plate is required, and therefore the thermal propagation is not interrupted. After heating started, the high temperature region expands similarly to those shown in Fig. 3. However, after reaching the top surface of the water, the hot region spread not only horizontally but also vertically in the oil region. Namely, the oil is heated by the water, and the high temperature region in the oil expands to the upper area as is the case with the water. In addition, the density of the bottom of the cell also changes indicating that the cell is heated and the temperature is increased.

These results show that the novel X-ray thermography is a powerful tool for the non-destructive detection and visualization of inner temperatures and thermal flows. Therefore, it is expected to enable quantitative analysis of thermal loadings in operating semiconductor power devices, electrical batteries, and motors for optimum thermal design. The coefficient of the thermal linear expansion of solid materials is smaller than that of liquid, for example, the coefficient of iron is 12 × 10^−6^/K and equivalent to 1/6 of that of water. However, it is expected that the high sensitivity of the imaging method enables the detection of the temperature of solid materials in 10 degrees of accuracy^16^. In addition, it will be possible to visualise the phonon dispersion and flow in thermal electric devices by applying a pump (heating by femto second laser) & probe (detecting using femto second pulse X-rays such as a free electron laser) method. For biomedical applications, quantitative analysis of antibody reaction for medicine and allergic substance can be performed from the view-point of thermal scale. A novel diagnostic method based on the relationship between inflammations, diseases, and thermals will be possible. Furthermore, an optimum freezing method can be developed by measuring the inside and outside temperature of foods. This method can be applied for novel cryopreservation without harming biomedical samples.

Practical use of the thermography requires shorter measurement times, increased spatial resolution, and an expanded dynamic range of density. The measurement time can be shorten by optimizing the X-ray energy and thinning the crystal wafer of the X-ray interferometer, which absorb X-ray intensity. Thinning the crystal wafer also provides another advantage in that it increases the spatial resolution, because the blurring of interference patterns in the wafer can be suppressed. By combining the thinning of the wafer and employing an X-ray imager with a high-spatial resolution, the spatial resolution can be reduced by less than 10 microns. The penetrating power of X-ray is proportional to the X-ray energy, and therefore thick samples that included heavy elements can be observed by using high-energy X-ray such as 35 keV^17^. We plan to implement the above improvements and perform an observation of power electrical devices in operation as the next step.

## Method

### Principle of X-ray thermography

Complex refractive index of material in hard X-ray region is given by

1 $$n=1-{\rm{\delta }}+{\rm{i}}{\rm{\beta }}$$

where δ and β are the real- and imaginary part of the index, and represented as

2 $$\begin{array}{c}\delta =\frac{{\lambda }^{2}{r}_{e}}{2\pi }\sum _{j}{m}_{j}\,({Z}_{j}+{{f}^{{\rm{^{\prime} }}}}_{j})\\ \beta =\frac{{\lambda }^{2}{r}_{e}}{2\pi }\sum _{j}\,{m}_{j}\,(-\,{{f}^{{\rm{^{\prime} }}{\rm{^{\prime} }}}}_{j})\end{array}$$

where λ, r_e_, and m_j_ are wavelength of X-ray, classical electron radius, number of atomics in unit cell, respectively, with f′ and f″ as the real and imaginary part of the anomalous dispersion of the atomic scattering factor. Furthermore, m_j_ is given by

3 $${m}_{j}=\frac{{N}_{A}\rho {x}_{j}}{{\sum }_{j}\,{x}_{j}{M}_{j}}$$

where, N_A_, ρ, x_j_, and M_j_ are the Avogadro constant, electron density, ratio of j-th element, and atomic number of j-th elements, respectively. Phase shift *dp* caused by a sample having a thickness of *t* is given by

4 $$dp=\frac{\delta t}{\lambda }2\pi .$$

However, the volume of material with changed temperature *dT* from its original value *V*_o_ can be approximated as

5 $$V={V}_{0}(1+cdT)$$

where *c* is the thermal expansion coefficient. Therefore, using a relation between volume and density $(\frac{d\rho }{dT}=-\,c{\rm{\rho }})$, the *dT* can be expressed using the detected phase-shift *dp* as

6 $$dT=-\,\frac{4\pi }{{\lambda }^{2}{r}_{e}{N}_{A}c\rho }dp$$

### XI imaging system and measurement conditions

Two- and three-dimensional observations were performed by using a phase-contrast imaging system fitted with an X-ray interferometer consisting of two-crystal blocks with two thin crystal wafers on it. The incident X-rays were divided, reflected, and recombined by the wafers, and two interference patterns were generated. Phase-shift caused by a sample was detected by the intensity changes in the interference pattern. Note that mechanical stability of the order of X-ray wavelength (sub-nrad) for the operation of the X-ray interferometer was achieved by a feedback system, rigid positional stages, and multiple hoods.

The interference pattern was detected by a fibre-coupled CCD imager. The X-rays were converted to visible light by a scintillator (GOS 30 μm), and transferred to a cooled CCD camera by optical fibre. The taper ratio of the fibre was 1:1.4, and the pixel size of the imager was 12.5 μm (CCD was 9 μm). The number of pixel was 4008 and 2650 in the horizontal and vertical direction. To obtain a quantitative phase map (distribution of phase shift), a fringe scanning method with three steps was applied. A phase-shifter made of an acrylic wedge was placed in path II, and moved vertically to obtain interference patterns at different phase relations to calculate quantitative phase-shift at each pixel. Monochromated X-rays (17.8 keV) were used for all observations. The number of projections of the phase-contrast computed tomography was set at 500 for 360 degrees. The exposure times for obtaining one interference pattern were 0.2 and 1 s for two- and three-dimensional observation.
